# Healthcare utilization prior to a diagnosis of young-onset Alzheimer’s disease: a nationwide nested case–control study

**DOI:** 10.1007/s00415-023-11974-x

**Published:** 2023-09-05

**Authors:** Line Damsgaard, Janet Janbek, Thomas Munk Laursen, Gunhild Waldemar, Christina Jensen-Dahm

**Affiliations:** 1 Department of Neurology, Danish Dementia Research Centre, Section 8008, Copenhagen University Hospital-Rigshospitalet, Blegdamsvej 9, 2100 Copenhagen, Denmark; 2 Department of Economics and Business Economics, National Centre for Register-based Research, Aarhus BSS, Aarhus University, Fuglesangs Allé 26-Building R, 8210 Aarhus V, Denmark; 3 Department of Clinical Medicine, University of Copenhagen, Blegdamsvej 3B, 2200 Copenhagen, Denmark

**Keywords:** Young-onset dementia, Alzheimer’s disease, Healthcare utilization, Early warning signs, Registry-based, Epidemiology

## Abstract

**Objective:**

Our aim was to identify changes in healthcare utilization prior to a young-onset Alzheimer’s disease diagnosis.

**Methods:**

In a retrospective incidence density matched nested case–control study using national health registers, we examined healthcare utilization for those diagnosed with young-onset Alzheimer’s disease in Danish memory clinics during 2016–2018 compared with age- and sex-matched controls. Negative binomial regression analysis produced contact rate ratios.

**Results:**

The study included 1082 young-onset Alzheimer’s disease patients and 3246 controls. In the year preceding diagnosis, we found increased contact rate ratios for all types of contacts except physiotherapy. Contact rate ratios for contacts with a general practitioner were significantly increased also > 1–5 and > 5–10 years before diagnosis. The highest contact rate ratios were for psychiatric emergency contacts (8.69, 95% CI 4.29–17.62) ≤ 1 year before diagnosis.

**Interpretation:**

Results demonstrate that young-onset Alzheimer’s disease patients have increased healthcare utilization from 5 to 10 years prior to diagnosis. Awareness of specific alterations in health-seeking behaviour may help healthcare professionals provide timely diagnoses.

**Supplementary Information:**

The online version contains supplementary material available at 10.1007/s00415-023-11974-x.

## Background

While Alzheimer’s disease (AD) is most often thought of as an affliction in the older population, AD is the most prevalent cause of dementia among both those with young-onset dementia [1] and those with late-onset dementia [2]. Young-onset AD (YOAD) thus affects individuals in the prime of their career with potentially devastating effects for both patients and their families, who may be financially dependent on the patient and may include children still living at home.

Increasing evidence suggests that AD pathology develops years and even decades before the onset of cognitive symptoms [3] and that cognitive changes can be detected well before onset of clinical AD [4]. A recent Norwegian retrospective study of 223 patients based on review of medical records found a substantial diagnostic delay in patients with YOAD with an average time lag of 3.4 years from onset of symptoms to first visit to their general practitioner (GP) and 5.5 years from symptom onset to diagnosis [5]. This highlights the need for improvement in making a timely diagnosis in this patient group. A timely diagnosis offers patients and caregivers the opportunity to obtain treatment to control symptoms and avoid medications that may worsen symptoms, as well as plan for the future and seek appropriate care [6]. This is especially true for those with YOAD due to changes in work ability and family roles. In YOAD, symptoms at time of diagnosis have been described to often differ from those seen in individuals with late-onset AD (LOAD) [7]. As the majority of research in the field of AD is focused on LOAD, there is a need for increased knowledge on the specific diagnostic challenges in young-onset dementia.

Understanding healthcare utilization patterns in the years preceding AD diagnosis may be a new piece in the puzzle of timely detection and may help to demonstrate whether these patients show vulnerability prior to diagnosis and, if so, for how long. The aim of this study was to describe patterns in healthcare utilization characteristic of the prodromal phase of YOAD and to identify any specific types of healthcare contacts with a more pronounced association than others.

## Methods

### Data source

Since 1968, all people living in Denmark have been registered in the Civil Registration System (CRS) with a unique 10-digit personal identification number. This number is used in all national registers, enabling accurate linkage between the registers [8]. Cases were identified from the Danish Quality Database for Dementia (DanDem), which was established in 2016. It is mandatory for every secondary healthcare facility which accepts referrals for dementia to enter information in DanDem upon completion of the diagnostic evaluation, including level of cognitive decline (normal cognition, mild cognitive impairment, or mild, moderate or severe dementia), aetiology, diagnostic investigations performed and results of cognitive tests. It thus follows that DanDem contains detailed data on every patient seen at a Danish memory clinic from 2016 onwards. Information on contacts with the primary healthcare sector was drawn from the National Health Service Registry (NHSR), which includes information on patients, providers and health services with national coverage since 1990 [8]. Information on contacts with the secondary healthcare sector was drawn from the Danish National Patient Registry (DNPR) and the Danish Psychiatric Central Research Register (DPCRR). DNPR and DPCRR contain information on all somatic and psychiatric hospital contacts since 1977 and 1969, respectively, with outpatient data added in 1995 [9, 10]. The Danish National Prescription Registry (DNPrR) was used to draw information on filled prescription medication used in the exclusion of potential controls [11].

### Study design and population

We conducted a retrospective incidence density matched nested case–control study. Cases were defined as all individuals diagnosed with YOAD in a Danish memory clinic during the years 2016–2018 and their matched controls were drawn randomly from the same nationwide cohort of all Danish residents.

#### Overall exclusion criteria

We excluded individuals with Down syndrome (International classification of diseases (ICD)-8: 759.3, ICD-10: Q90) and mental retardation (ICD-8: 311–315, ICD-10: F70–F79) as morbidity prior to AD diagnosis for this patient group would require other surrogate measures of morbidity than those addressed in the present study.

To ensure completeness of data, cases and controls must have lived in Denmark throughout the 10-year retrospective period. Controls were not eligible if they had a diagnosis of dementia prior to index date. All exclusion criteria can be seen in Online Resource Table S1.

#### Case definition

By convention, YOAD is defined as dementia due to AD with symptom onset before age 65 years. Due to the often long time lag between symptom onset and diagnosis [5], all patients diagnosed with mild cognitive impairment (MCI) or dementia due to AD before age 70 years were included. Thus, cases were identified from DanDem as individuals with a first diagnosis of MCI or dementia due to AD from start of register (2016) through 2018 aged < 70 years at time of diagnosis. Index date was the date when the person was diagnosed with AD.

#### Controls

Cases were matched 1:3 with controls on birthdate and sex using incidence density matching. Controls were randomly selected from the entire population with the following criteria: (1) no entry in DanDem before index date (i.e. not referred to or seen at a memory clinic), (2) no MCI or dementia diagnosis in DNPR or DPCRR before index date and (3) no redeemed prescription for antidementia medication in DNPrR before index date (ICD codes for identification of dementia diagnosis and anatomical therapeutic chemical (ATC) codes for dementia medication can be found in Online Resource Table S1).

### Definition of healthcare contacts

The exposures of interest were the number and type of contacts within the primary or secondary healthcare sector. In the secondary healthcare data (somatic and psychiatric contacts), any contact with an ICD code indicating MCI or dementia (Online Resource Table S1) was censored for cases in the 6 months prior to index date to avoid counting those contacts that were part of the diagnostic process.

#### Primary care

Primary care contacts were defined as a record in NHSR in one of the following categories: face-to-face contact with a GP, a telephone or email contact with a GP, any type of contact with a private practice medical specialist, or any contact with a physiotherapist or clinical psychologist by referral. There is no public record of visits to a physiotherapist or psychologist if a patient chooses to visit one without referral from the public sector, and thus, such visits are not included in the contact counts. For GP contacts, we also combined face-to-face and telephone/email contacts into ‘any GP contact’.

#### Secondary care—somatic contacts

In this study, the term ‘somatic’ is used to refer to non-psychiatric disorders, encompassing physical health issues distinct from psychological or psychiatric conditions. Somatic contacts were defined as a record in DNPR in one of the following categories: inpatient admission, outpatient contact or emergency contact. For inpatient admissions, if a patient was discharged and re-admitted in < 24 h, this was considered a continuation of the same admission and was counted as one admission. Outpatient contacts were defined as every time a patient was present at a hospital for an outpatient consultation.

#### Secondary care—psychiatric contacts

Psychiatric contacts were defined as a record in DPCRR in one of the following categories: inpatient admission, outpatient contact or emergency contact. Inpatient admissions were defined the same as described above. As individual visits within a longer follow-up are not available for psychiatric outpatient contacts, these contacts were counted as numbers of follow-up processes rather than individual visits.

### Covariates

Analyses were adjusted for age, sex, highest attained educational level at age 40 years (or at time of diagnosis, whichever came first) and civil status at index date.

### Statistical analysis

#### Main analysis

We analysed associations between all the above listed types of healthcare contacts except psychiatric outpatient contacts and dementia using a negative binomial regression analysis, which produces an odds ratio. Given the use of incidence density matching, this can be interpreted as an incidence rate ratio [12], or in this case, a contact rate ratio (CRR). CRRs were calculated for three time intervals: 10-> 5 years prior to index date, 5-> 1 years prior to index date and ≤ 1 year prior to index date. Robust variance estimation was applied to account for matching clusters and correlation between observations for the same person in different periods using the vce(cluster) function in STATA to allow for intragroup correlation.

As number of individual visits were not available for psychiatric outpatient data, we instead measured the proportion of persons in our study with a psychiatric outpatient contact during the 10 years prior to dementia diagnosis by a cumulative incidence function.

Analyses were adjusted for the listed covariates.

#### Sensitivity analysis

In a sensitivity analysis, cases were stratified on disease severity. Here, patients were grouped as either MCI/mild dementia or moderate/severe dementia at time of diagnosis. Analyses were then performed as described above, comparing the disease severity groups with their respective matched controls. Furthermore, sensitivity analyses were performed stratifying cases by age at diagnosis and sex.

Data management was performed using SAS 9.4 software. Negative binomial regression analyses were performed using STATA/MP 17.0 software.

## Results

**Fig. 1:**
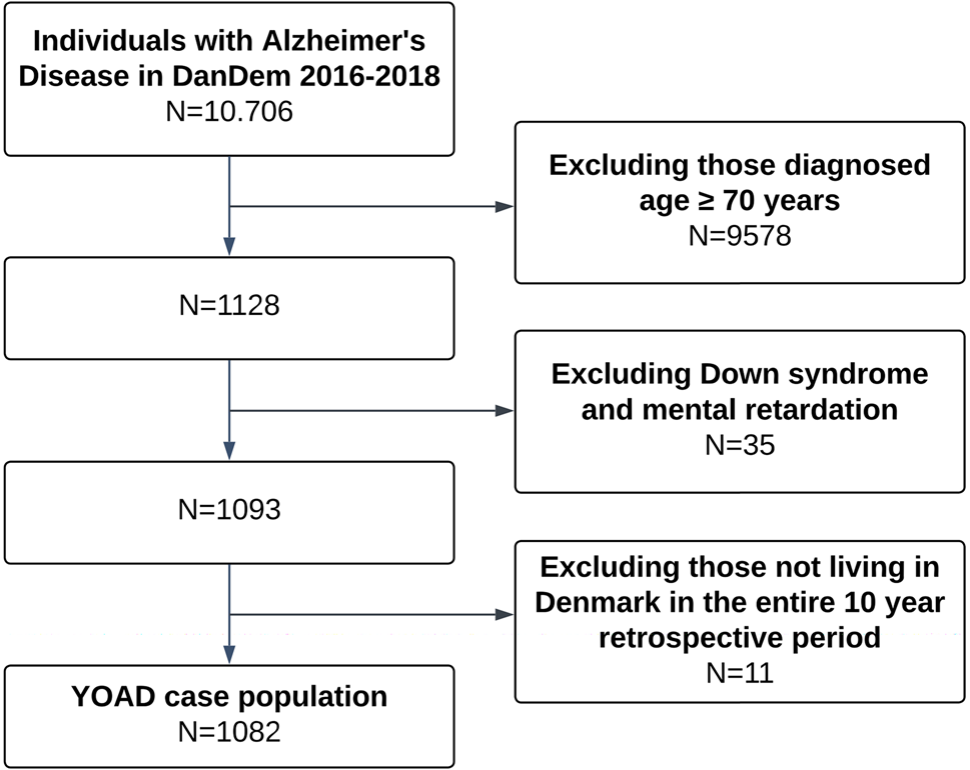
Flowchart of case selection. *DanDem* Danish Quality Database for Dementia, *YOAD* young-onset Alzheimer’s disease

There were 10,706 individuals with a diagnosis of AD in DanDem between 2016 and 2018. Of these, 1128 were diagnosed before age 70 years. After exclusions, there were 1082 cases (Fig. 1). The mean age at time of diagnosis was 64.5 years (range 29.2–69.9 years, Table 1), and 58% of the patients were female. At the initial assessment in a memory clinic, mean Mini-Mental State Examination (MMSE) score was 20.8 points and mean Addenbrooke’s cognitive examination (ACE) score was 65.4 points. Among the cases, 60% were diagnosed with either MCI or mild dementia at time of diagnosis, and the remaining 40% were diagnosed with moderate/severe dementia. Cases and controls were similar in terms of education and civil status.

**Table 1:** Baseline characteristics of study population

	Cases *n* = 1082	Controls *n* = 3246
Age at index date, mean years (sd) [range]	64.5 (5.1) [29.2–69.9]	64.5 (5.1) [29.2–69.9]
Sex, male/female, *n* (%)	456 (42%)/626 (58%)	1368 (42%)/1878 (58%)
Level of cognitive decline at diagnosis, *n* (%)		
Mild cognitive impairment	39 (4%)	–
Mild dementia	611 (56%)	–
Moderate dementia	346 (32%)	–
Severe dementia	86 (8%)	–
Cognitive examination scores at first visit^a^, mean (sd)		
MMSE	20.8 (5.6)	–
ACE	65.4 (15.2)	–
Educational attainment at age 40, *n* (%)		
Low	734 (68%)	2218 (69%)
Medium	239 (22%)	679 (21%)
High	62 (6%)	189 (6%)
Unknown	47 (4%)	147 (5%)
Civil status at index date, *n* (%)		
Married	685 (63%)	2030 (63%)
Divorced	197 (18%)	568 (18%)
Widowed	86 (8%)	247 (8%)
Never married	100 (9%)	357 (11%)
Unknown	14 (1%)	31 (1%)
Individuals with ≥ 1 contact in primary care, *n* (%)^b^		
General practice	1082 (100%)	3227 (99%)
Psychology	61 (6%)	75 (2%)
Physiotherapy	502 (46%)	1.510 (47%)
Private practice medical specialist	930 (86%)	2.674 (82%)
Individuals with ≥ 1 contact in secondary care, *n* (%)^b^		
Somatic inpatient	658 (61%)	1720 (53%)
Somatic outpatient	1026 (95%)	2829 (87%)
Somatic emergency	687 (37%)	1716 (53%)
Psychiatric inpatient	61 (6%)	75 (2%)
Psychiatric outpatient	155 (14%)	127 (4%)
Psychiatric emergency	73 (7%)	82 (3%)

Where percentages do not add up to 100%, this is due to rounding up/down

^a^MMSE: mini-mental state examination (reliable information for 988 YOAD patients). ACE: Addenbrooke’s Cognitive Examination (reliable information for 660 YOAD patients)

^b^Percentages in this section of the table represent the number of people with at least 1 contact during the 10-year period out of the total case/control population

**Fig. 2:**
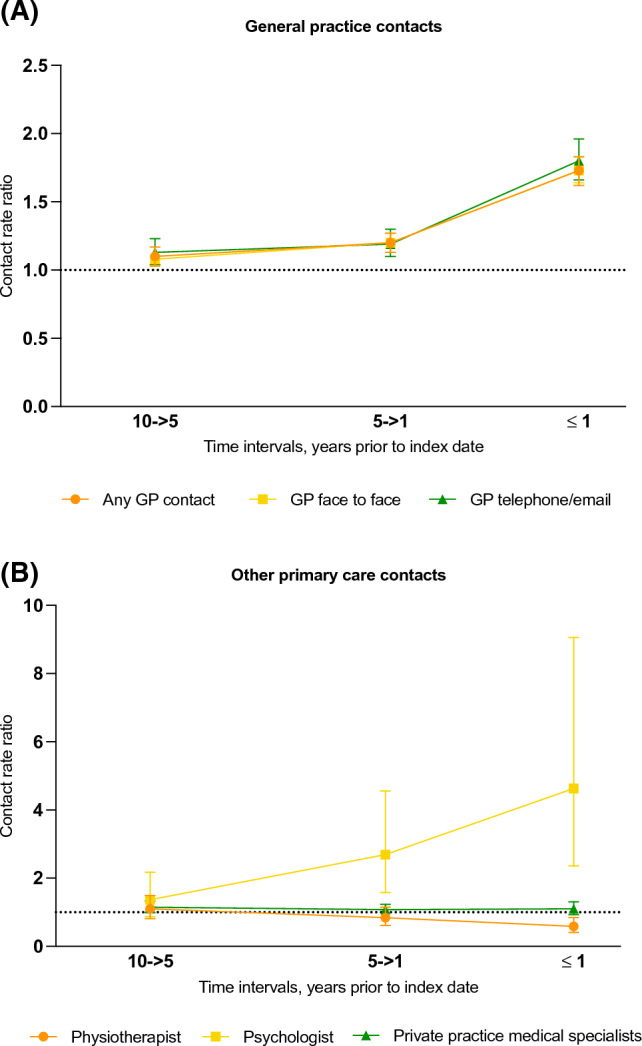
Contact rate ratios, primary care contacts. Contact rate ratios (CRRs) are plotted for young-onset Alzheimer’s disease patients for contacts with a general practitioner (**A**) and other secondary healthcare contacts (**B**). Error bars represent 95% confidence intervals. The CRRs presented are adjusted for age, sex, highest attained educational level at age 40 (or at time of diagnosis, whichever came first) and civil status at index date. Note that the Y-axis have differing values for A and B. Unadjusted estimates are presented in Online Resource Table S3 and adjusted estimates are presented in Table 2. *GP* general practitioner

**Fig. 3:**
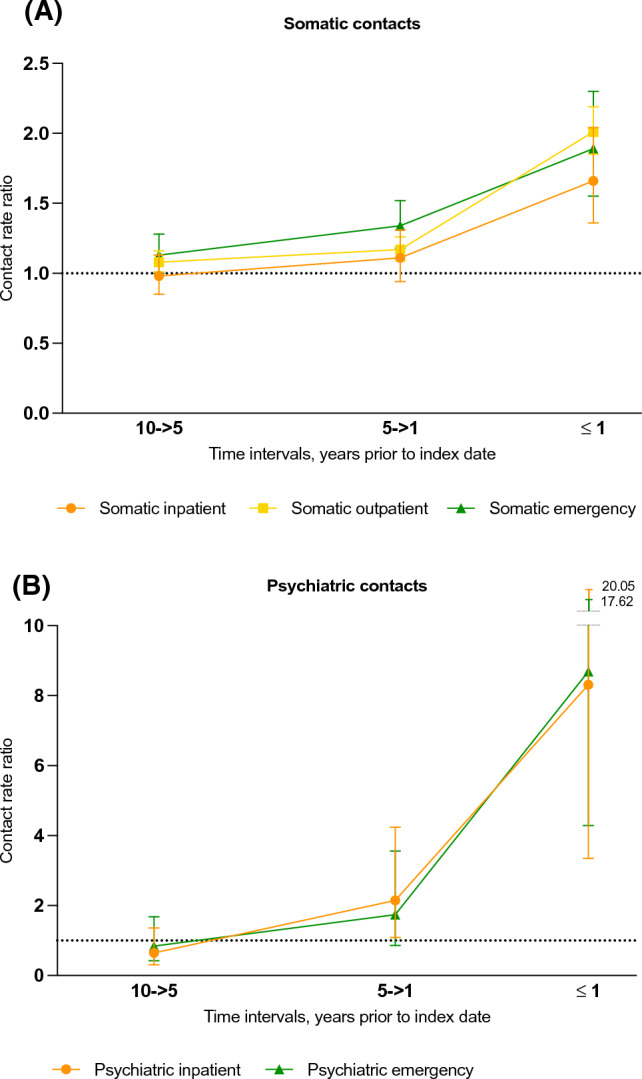
Contact rate ratios, secondary care contacts. Contact rate ratios (CRRs) are plotted for young-onset Alzheimer’s disease patients for somatic healthcare contacts (**A**) and psychiatric healthcare contacts (**B**). Error bars represent 95% confidence intervals. The CRRs presented are adjusted for age, sex, highest attained educational level at age 40 (or at time of diagnosis, whichever came first) and civil status at index date. Note that the Y-axis have differing values for A and B. Unadjusted estimates are presented in Online Resource Table S3 and adjusted estimates are presented in Table 2

Figures 2, 3 and Table 2 show the adjusted CRR for YOAD cases compared to controls (contact rates and unadjusted CRRs in Online Resource Tables S2 and S3). In the year prior to index date, CRRs for all investigated types of contacts were significantly increased except for private practice medical specialist contacts (insignificant CRRs) and contacts to a physiotherapist by referral (significantly decreased, CRR 0.59, 95% CI 0.41–0.85). Across all types of GP contacts, CRRs were significantly increased throughout the 10-year retrospective period and increased steadily to the highest level in the time interval immediately preceding dementia diagnosis where YOAD patients had a 73% higher contact rate (any type) than their matched controls (CRR 1.73, 95% CI 1.64–1.83).

**Table 2:** Consultation rate ratios by type of healthcare contact, fully adjusted

Consultation rate ratios (CRR)	Adjusted*		
	CRR	95% CI	*P* value
Primary care			
Any GP contact			
10-> 5 years prior to index date	1.10	1.03–1.17	0.003
5-> 1 years prior to index date	1.20	1.13–1.27	≤ .00001
≤ 1 year prior to index date	1.73	1.64–1.83	≤ .00001
GP Face-to-face contacts			
10-> 5 years prior to index date	1.08	1.01–1.14	0.021
5-> 1 years prior to index date	1.20	1.13–1.28	≤ .00001
≤ 1 year prior to index date	1.67	1.58–1.76	≤ .00001
GP telephone/email contacts			
10-> 5 years prior to index date	1.13	1.04–1.23	0.004
5-> 1 years prior to index date	1.19	1.10–1.30	≤ .00001
≤ 1 year prior to index date	1.80	1.66–1.96	≤ .00001
Physiotherapist			
10-> 5 years prior to index date	1.10	0.81–1.49	0.560
5-> 1 years prior to index date	0.84	0.61–1.15	0.279
≤ 1 year prior to index date	0.59	0.41–0.85	0.005
Psychologist			
10-> 5 years prior to index date	1.37	0.87–2.18	0.178
5-> 1 years prior to index date	2.69	1.58–4.56	≤ .00001
≤ 1 year prior to index date	4.63	2.36–9.06	≤ .00001
Private practice medical specialist			
10-> 5 years prior to index date	1.15	0.99–1.33	0.069
5-> 1 years prior to index date	1.08	0.95–1.23	0.240
≤ 1 year prior to index date	1.10	0.93–1.31	0.277
Secondary care			
Somatic inpatient admissions			
10-> 5 years prior to index date	0.98	0.85–1.13	0.797
5-> 1 years prior to index date	1.11	0.94–1.31	0.217
≤ 1 year prior to index date	1.66	1.36–2.04	≤ .00001
Somatic outpatient contacts			
10-> 5 years prior to index date	1.08	1.01–1.16	0.028
5-> 1 years prior to index date	1.17	1.09–1.26	≤ .00001
≤ 1 year prior to index date	2.01	1.85–2.19	≤ .00001
Somatic emergency admissions			
10-> 5 years prior to index date	1.13	1.00–1.28	0.045
5-> 1 years prior to index date	1.34	1.18–1.52	≤ .00001
≤ 1 year prior to index date	1.89	1.55–2.30	≤ .00001
Psychiatric inpatient admissions			
10-> 5 years prior to index date	0.65	0.31–1.36	0.255
5-> 1 years prior to index date	2.15	1.09–4.24	0.028
≤ 1 year prior to index date	8.31	3.35–20.60	≤ .00001
Psychiatric emergency admissions			
10-> 5 years prior to index date	0.84	0.42–1.68	0.618
5-> 1 years prior to index date	1.74	0.86–3.56	0.125
≤ 1 year prior to index date	8.69	4.29–17.62	≤ .00001

*GP* general practitioner, *CRR* contact rate ratio, *CI* confidence interval

*Adjusted for age, sex, highest attained educational level at age 40 (or at time of diagnosis, whichever came first) and civil status at index date. CRRs are presented in time intervals prior to the date of dementia diagnosis for cases and compared to the control group of individuals free of dementia at index date. Unadjusted estimates are presented in Supplementary Table S2

**Fig. 4:**
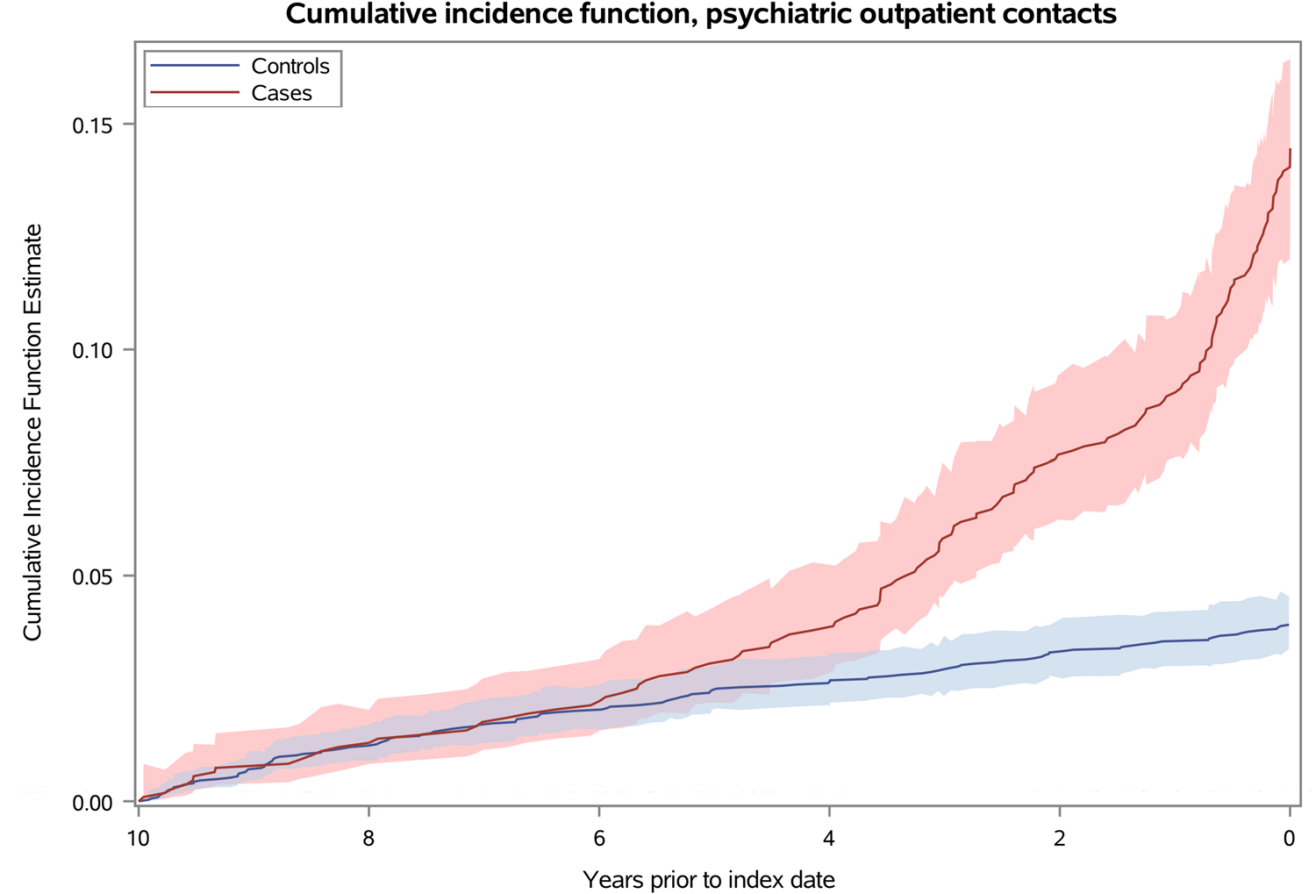
Cumulative incidence function, psychiatric outpatient contacts. The figure shows the cumulative incidence of psychiatric outpatient contacts for dementia cases and controls, respectively, over a 10-year period prior to the index date, which is the date of diagnosis for the dementia cases. The x-axis represents time in years, and the y-axis represents the cumulative incidence of psychiatric outpatient contacts

The largest increase in CRR was for psychologist and psychiatric contacts in the year immediately preceding diagnosis, though confidence intervals are wide as these estimates are based on relatively few contacts, partly owing to the fact that the interval immediately prior to diagnosis is much shorter than the other intervals. The CRR for psychology contacts was 4.63 (95% CI 2.36–9.06). For psychiatric inpatient and emergency contacts, CRR in the year prior to diagnosis was increased eightfold compared to controls (psychiatric inpatient admissions: CRR 8.31, 95% CI 3.35–20.60, psychiatric emergency contacts: CRR 8.69, 95% CI 4.29–17.62). A similar tendency can be seen in the cumulative incidence function for psychiatric outpatient contacts (Fig. 4), where the cumulative incidence is significantly higher for cases compared to controls from 3.5 years prior to diagnosis.

Apart from the GP contacts, CRRs in the > 1–5 and > 5–10 years prior to diagnosis were generally either insignificant or only minorly increased. The only other clear change in the 5-> 1 year interval was for psychologist contacts (CRR 2.69, 95% CI 1.58–4.56).

For those with moderate/severe dementia, the increase in contact rates generally occurred earlier than for those with MCI/mild dementia at time of diagnosis. For GP contacts, those with moderate/severe dementia had an increase in contact rates already 5–10 years prior, whereas the increase only occurred from 1 to 5 years prior for those with MCI/mild dementia (Online Resource Table S4). For psychologist contacts, those with moderate/severe dementia had the highest CRR in the 5-> 1 year interval (3.59, 95% CI 1.77–7.31), whereas for those with MCI/mild dementia the highest CRR was found in the year immediately preceding diagnosis (CRR 5.17, 95% CI 2.32–11.50). The increased CRR for psychiatric contacts found in the main analysis was largely driven by those with moderate/severe dementia, for whom CRRs were highly increased in the year preceding diagnosis with a CRR for psychiatric emergency contacts of 23.76 (95% CI 7.59–74.38, Online Resource Table S5). A sensitivity analysis stratifying YOAD patients by age and sex yielded similar results as in the main analysis (Online Resource Table S6).

## Discussion

This nested case–control study found that patients with YOAD generally have a higher healthcare utilization than their age- and sex-matched controls in the year preceding diagnosis. The increase in healthcare utilization could be traced back potentially as long as 10 years for GP contacts. Only 60% of the patients in our study were classified as having MCI or mild dementia at the time of diagnosis in a memory clinic, while the remaining 40% were already at the moderate/severe stage of the disease at diagnosis. In the sensitivity analysis examining healthcare utilization in YOAD patients according to disease severity at time of diagnosis, CRRs were generally higher and increased earlier for those with moderate/severe dementia than for those with MCI/mild dementia, indicating a window of opportunity for a more timely diagnosis.

To our knowledge, this is the first study to examine healthcare utilization patterns prior to dementia diagnosis in YOAD patients, while there have been some studies examining healthcare utilization prior to a diagnosis of late-onset dementia or not stratifying by age [13–27]. The majority of studies on healthcare utilization prior to a dementia diagnosis examined only the time period 1–2 years prior to diagnosis and found increased healthcare utilization/cost throughout the study periods [18, 21–23]. A few studies examined 2–3 years prior to diagnosis and found increased healthcare utilization only immediately preceding diagnosis [24, 25]. The longest examined time prior to dementia diagnosis was in the study by Ramakers et al. [17], who examined GP contacts in the five years prior to dementia diagnosis and found an increase throughout the entire study period. A study from the UK utilized data from both general practitioners and hospital contacts, wherein they found healthcare utilization significantly increased in patients with LOAD throughout the 3-year study period before diagnosis, especially in the 6 months immediately preceding diagnosis [13]. The authors suggest this may reflect the diagnostic process or may reflect that those with AD are less able to maintain healthy habits and compliance with pharmacotherapy.

In a population of patients with AD and related disorders, Desai et al. examined whether healthcare resource use was related to disease severity at the time of initial cognitive assessment and found an increase in healthcare utilization years before dementia diagnosis, which increased with disease severity at the time of assessment [20]. Although they measured disease severity by MMSE or Montreal Cognitive Assessment (MoCA) score, whereas we determined disease severity according to ICD-10 research criteria for dementia staging, our results are in line.

Although these previous studies focused on LOAD, the longest examined period was 5 years retrospectively, and the methodology differs, the results are in line with ours. Thus, these findings on YOAD are consistent with the findings from studies on LOAD; healthcare utilization is increased several years before dementia diagnosis, especially immediately preceding diagnosis, and is influenced by disease severity. This study was designed to explore the overall contact rates, and further exploration of the reasons behind these contacts should be done in a suitably designed future study.

Our results add to these previous studies by providing additional insight into the younger AD patients whose healthcare utilization patterns have not previously been described. Those with moderate/severe dementia seem to exhibit signs of vulnerability throughout the entire study period, likely suggesting that these patients have had dementia symptoms and deteriorating health for a long period, rather than a rapid disease course. Furthermore, these patients have a drastically increased CRR for both psychiatric inpatient and emergency admissions, likely portraying how immensely difficult it can be to recognize dementia symptoms in young patients, which may lead to misinterpretation of symptoms. Though confidence intervals for these associations are very wide, even if the true value of the CRRs is near the lower bounds of the confidence intervals, this is still a large increase compared to the controls. Dementia symptoms could be misinterpreted as psychiatric diseases such as stress, anxiety or depression, or these diseases may occur comorbidly [28], blurring the picture of the dementia symptoms and hindering timely diagnosis. It also seems likely that dementia symptoms are overlooked entirely until patients are admitted with delirium or behavioural and psychological symptoms of dementia, all of which occur more frequently and more severely further along the disease course [29, 30]. Similarly, the timing of the peak in psychologist contacts differs according to disease severity with those with MCI/mild dementia having the highest CRR for psychologist contacts in the year prior to diagnosis. For those with moderate/severe dementia, this seems to happen earlier, perhaps reflecting a timing of when symptoms begin to markedly impact functioning of the patients at work or at home. The explanation for these findings is likely found in a long time lag between symptom onset and diagnosis. There may also be an unwillingness among young patients with cognitive complaints in being referred to a memory clinic or a lack of disease insight which may also add to the diagnostic delay.

Another possible explanation for the overall increased healthcare utilization long before diagnosis could be that comorbidities such as diabetes, hypertension, cardiovascular disease and a multitude of other chronic diseases may lead to frequent GP visits. These are also risk factors for developing dementia or for a more rapid cognitive decline [31], and the increased contact rate and dementia diagnosis may thus share a common cause. Failure to comply with planned treatment regimens due to cognitive decline could lead to a general health deterioration, which could also lead to increased healthcare utilization. If healthy habits are increasingly compromised, this could also explain why we see a decreased use of physiotherapy visits by referral prior to diagnosis in the present study.

Our study had several limitations. The study design is vulnerable to detection bias; those who see a medical doctor frequently are more likely to be referred to a memory clinic and receive a diagnosis upon experiencing symptoms than those who do not regularly see a doctor, whereas symptoms of dementia in those who do not frequently see a doctor may go unnoticed. It is noteworthy, though, that only 60% of cases were diagnosed in the early stages of the disease, suggesting that seeing a doctor frequently is not necessarily sufficient to receive a timely diagnosis.

While censoring of contacts related to the diagnostic process in the memory clinic was attempted, it is not possible to completely remove all such contacts if not specifically marked with a dementia diagnosis. Furthermore, the primary care registers used does not contain reasons for contacts and censoring could not be done. For these reasons, GP and outpatient contacts in the time interval immediately before diagnosis should be interpreted taking this into account. This should not, however, majorly influence results further back in time.

Another limitation is the unavailability of genetic information in the registers. This is especially relevant for those with YOAD, where genetic mutations contribute to a larger number of AD cases than what is seen in LOAD [32].

A major strength of the present study is the use of nationwide healthcare registers. The majority of previous studies relied on claims/insurance data [18–27], which may result in a tendency to favour a healthier population [33]. In Denmark, the health services examined were, with few exceptions, free of charge, and data on healthcare contacts were drawn from the Danish healthcare registers which contain detailed, reliable and complete data on all contacts with a GP or hospital, limiting problems with selection bias. Thus, there is equal access to healthcare services for all and virtually full data coverage. However, for physiotherapy and psychologist contacts we only had data for those referred within the Danish public sector. Physiotherapists and clinical psychologists can also be seen using out-of-pocket payment or a private health insurance, and thus, these results should be interpreted with caution.

In conclusion, this study demonstrated an altered healthcare utilization pattern across all 12 investigated types of contacts in the year immediately preceding diagnosis. GP contacts were significantly increased throughout the entire 10-year study period, particularly for those with moderate/severe dementia at time of diagnosis. There was a substantial increase especially for psychiatric contacts, showcasing the need for a more timely diagnosis, which could perhaps decrease avoidable hospitalizations due to psychiatric symptoms of yet undiagnosed dementia. Increased healthcare utilization in young patients with cognitive complaints could potentially be an early warning sign of AD, though further research into such early warning signs is needed to paint a full picture of the earliest phases of YOAD.

### Supplementary Information

Below is the link to the electronic supplementary material.

Supplementary file1 (PDF 411 KB)

## Data Availability

All of the data used in this study are derived from the Danish National and Public Health registries. These data are collected and stored by the relevant authorities and cannot be made public or accessed by unauthorized parties. Access to such data is given via standard rules and regulations of data access outlined by the Danish Data Protection Agency and Danish Health Data Authority.
