# Supplementary material for: Healthcare utilization prior to a diagnosis of young-onset Alzheimer’s disease: a nationwide nested case–control study
Source: J Neurol. 2023 Sep 5;270(12):6093–102. doi: 10.1007/s00415-023-11974-x (PMC10632232; doi:10.1007/s00415-023-11974-x)
Supplement: Supplementary file 1 — Supplementary file1 (PDF 411 KB) [file 415_2023_11974_MOESM1_ESM.pdf]

Supplementary

Healthcare utilization prior to a diagnosis of young onset Alzheimer's disease: A nationwide nested case-control study

Damsgaard L, Janbek J, Laursen TM, Waldemar G, Jensen-Dahm C. Journal of Neurology

Correspondence: Line Damsgaard, MD

Danish Dementia Research Centre, Section 8008, Department of Neurology, Copenhagen University Hospital - Rigshospitalet, Copenhagen, Denmark

Email: line.damsgaard@regionh.dk

Contents:

Table S1. Exclusion criteria

Table S2. Contacts rates for primary and secondary care contacts

Table S3. Contact rate ratios for primary and secondary care contacts, unadjusted

Table S4. Sensitivity analysis – Contact rate ratios by level of cognitive decline at diagnosis in primary care

Table S5. Sensitivity analysis – Contact rate ratios by level of cognitive decline at diagnosis in secondary care

Table S6. Sensitivity analysis - Contact rate ratios by type of healthcare contact, stratified by age and sex

Table S1. Exclusion criteria

	Cases	Controls
**Developmental disorders and mental retardation**<br>ICD-8: 311-315, 759.3, ICD-10: F70-F79, Q90	X	X
**Not living in Denmark in 10-year retrospective period**	X	X
**Dementia or mild cognitive impairment diagnosis**<br>ICD-8: 290.09-11, 290.18-19, 293.09-19, ICD 10: F00.0-00.9, F01.0-01.9, F02.0-F02.8, F03.9, F04.0-F04.9, F06.7 G30.0-G30.9, G31.0A, G31.0B, G31.8, G31.8E, G31.9	*	X
**Dementia medication**<br>ATC code N06DA02-4, N06DX01		X
**Entry in DanDem**		X

* ICD-codes used for censoring contacts 6 months before dementia diagnosis for cases

Abbreviations: ICD: International classification of diseases, ATC: Anatomical Therapeutic Chemical, DanDem: Danish Dementia Quality Database.

Table S2. Contact rates for primary and secondary care contacts

Primary care	Contact rates per person-year		Secondary care	Contact rates per person-year	
**Any GP contact**	**Cases**	**Controls**	**Somatic inpatient admissions**	**Cases**	**Controls**
10->5 years prior to index date	41.33	37.60	10->5 years prior to index date	0.68	0.71
5->1 years prior to index date	38.24	31.83	5->1 years prior to index date	0.77	0.70
≤1 year prior to index date	14.42	8.37	≤1 year prior to index date	0.36	0.22
**GP Face to face contacts**			**Somatic outpatient contacts**		
10->5 years prior to index date	22.89	21.33	10->5 years prior to index date	2.92	2.67
5->1 years prior to index date	21.35	17.85	5->1 years prior to index date	2.87	2.50
≤1 year prior to index date	7.64	4.65	≤1 year prior to index date	1.38	0.71
**GP telephone/email contacts**			**Somatic emergency admissions**		
10->5 years prior to index date	18.44	16.28	10->5 years prior to index date	0.64	0.57
5->1 years prior to index date	16.90	13.98	5->1 years prior to index date	0.68	0.51
≤1 year prior to index date	6.79	3.72	≤1 year prior to index date	0.27	0.14
**Physiotherapist**			**Psychiatric inpatient admissions**		
10->5 years prior to index date	9.50	8.10	10->5 years prior to index date	0.03	0.03
5->1 years prior to index date	7.05	7.89	5->1 years prior to index date	0.05	0.04
≤1 year prior to index date	1.40	2.11	≤1 year prior to index date	0.04	0.01
**Psychologist**			**Psychiatric emergency admissions**		
10->5 years prior to index date	0.36	0.36	10->5 years prior to index date	0.04	0.04
5->1 years prior to index date	0.57	0.24	5->1 years prior to index date	0.05	0.04
≤1 year prior to index date	0.24	0.06	≤1 year prior to index date	0.06	0.01
**Private practice medical specialist**					
10->5 years prior to index date	5.57	4.99			
5->1 years prior to index date	4.72	4.43			
≤1 year prior to index date	1.26	1.18			

Contact rates are presented in time intervals prior to index date (date of diagnosis for cases) for cases and controls separately by type of contact.
Abbreviations: GP: general practitioner

Table S3. Contact rate ratios for primary and secondary care contacts, unadjusted

Primary care	Unadjusted			Secondary care	Unadjusted		
**Any GP contact**	**CRR**	**95 % CI**	**P-value**	**Somatic inpatient admissions**	**CRR**	**95 % CI**	**P-value**
10->5 years prior to index date	1.10	1.03-1.17	0.003	10->5 years prior to index date	0.96	0.83-1.10	0.540
5->1 years prior to index date	1.20	1.13-1.28	≤.00001	5->1 years prior to index date	1.10	0.93-1.29	0.268
≤1 year prior to index date	1.72	1.62-1.83	≤.00001	≤1 year prior to index date	1.63	1.33-2.01	≤.00001
**GP Face to face contacts**				**Somatic outpatient contacts**			
10->5 years prior to index date	1.07	1.01-1.14	0.023	10->5 years prior to index date	1.09	1.02-1.17	0,011
5->1 years prior to index date	1.20	1.13-1.27	≤.00001	5->1 years prior to index date	1.15	1.07-1.23	≤.00001
≤1 year prior to index date	1.64	1.55-1.74	≤.00001	≤1 year prior to index date	1.92	1.78-2.08	≤.00001
**GP telephone/email contacts**				**Somatic emergency admissions**			
10->5 years prior to index date	1.13	1.04-1.24	0.005	10->5 years prior to index date	1.12	0.99-1.27	0.071
5->1 years prior to index date	1.21	1.11-1.32	≤.00001	5->1 years prior to index date	1.33	1.17-1.52	≤.00001
≤1 year prior to index date	1.82	1.67-1.99	≤.00001	≤1 year prior to index date	1.90	1.56-2.33	≤.00001
**Physiotherapist**				**Psychiatric inpatient admissions**			
10->5 years prior to index date	1.17	0.45-0.97	0.310	10->5 years prior to index date	0.76	0.38-1.50	0.424
5->1 years prior to index date	0.89	0.66-1.21	0.465	5->1 years prior to index date	1.45	0.57-3.65	0.433
≤1 year prior to index date	0.67	0.86-1.60	0.035	≤1 year prior to index date	6.50	2.16-19.60	0.001
**Psychologist**				**Psychiatric emergency admissions**			
10->5 years prior to index date	0.98	0.65-1.46	0.917	10->5 years prior to index date	1.01	0.52-1.96	0.980
5->1 years prior to index date	2.33	1.56-3.49	≤.00001	5->1 years prior to index date	1.23	0.53-2.81	0.631
≤1 year prior to index date	4.32	2.47-7.57	≤.00001	≤1 year prior to index date	6.77	3.05-15.04	≤.00001
**Private practice medical specialist**							
10->5 years prior to index date	1.12	0.97-1.28	0.119				
5->1 years prior to index date	1.07	0.94-1.21	0.312				
≤1 year prior to index date	1.07	0.91-1.25	0.424				

CRRs are presented in time intervals prior to the date of dementia diagnosis for cases and compared to the control group of individuals free of dementia at index date.

Abbreviations: GP: general practitioner, CRR: contact rate ratio, CI: confidence interval.

Table S4. Sensitivity analysis – Contact rate ratios by level of cognitive decline at diagnosis in primary care

Primary care	MCI / Mild dementia						Moderate / severe dementia					
	Unadjusted			Adjusted			Unadjusted			Adjusted		
**Any GP contact**	**CRR**	**95 % CI**	**P-value**	**CRR**	**95 % CI**	**P-value**	**CRR**	**95 % CI**	**P-value**	**CRR**	**95 % CI**	**P-value**
10->5 years prior to index date	1.01	0.94-1.09	0.801	1.03	0.95-1.11	0.484	1.23	1.11-1.36	≤.00001	1.20	1.09-1.32	≤.00001
5->1 years prior to index date	1.14	1.06-1.23	0.001	1.16	1.08-1.25	≤.00001	1.29	1.17-1.43	≤.00001	1.26	1.14-1.39	≤.00001
≤1 year prior to index date	1.60	1.49-1.72	≤.00001	1.65	1.54-1.76	≤.00001	1.91	1.73-2.12	≤.00001	1.87	1.70-2.07	≤.00001
**GP Face to face contacts**												
10->5 years prior to index date	1.01	0.94-1.10	0.728	1.03	0.95-1.12	0.417	1.16	1.06-1.28	0.002	1.14	1.04-1.26	0.007
5->1 years prior to index date	1.18	1.09-1.27	≤.00001	1.21	1.12-1.30	≤.00001	1.22	1.11-1.35	≤.00001	1.21	1.10-1.33	≤.00001
≤1 year prior to index date	1.65	1.54-1.77	≤.00001	1.70	1.59-1.82	≤.00001	1.63	1.48-1.80	≤.00001	1.62	1.47-1.79	≤.00001
**GP telephone/email contacts**												
10->5 years prior to index date	1.00	0.91-1.11	0.928	1.02	0.93-1.13	0.670	1.32	1.14-1.52	≤.00001	1.29	1.12-1.48	≤.00001
5->1 years prior to index date	1.09	0.98-1.22	0.106	1.11	1.00-1.23	0.046	1.38	1.20-1.59	≤.00001	1.32	1.15-1.52	≤.00001
≤1 year prior to index date	1.54	1.38-1.71	≤.00001	1.57	1.42-1.75	≤.00001	2.26	1.97-2.60	≤.00001	2.19	1.91-2.50	≤.00001
**Physiotherapist**												
10->5 years prior to index date	1.11	0.77-1.61	0.563	1.03	0.72-1.47	0.871	1.24	0.75-2.05	0.398	1.20	0.76-1.89	0.428
5->1 years prior to index date	0.92	0.64-1.33	0.669	0.85	0.60-1.19	0.345	0.85	0.51-1.44	0.552	0.81	0.50-1.32	0.394
≤1 year prior to index date	0.66	0.40-1.07	0.093	0.59	0.38-0.92	0.021	0.68	0.37-1.24	0.205	0.60	0.34-1.05	0.074
**Psychologist**												
10->5 years prior to index date	1.15	0.68-1-95	0.608	1.43	0.78-2.61	0.250	0.77	0.41-1.44	0.411	1.75	0.75-4.09	0.198
5->1 years prior to index date	2.55	1.51-4.29	≤.00001	2.65	1.31-5.33	0.007	1.99	1.08-3.69	0.028	3.59	1.77-7.31	≤.00001
≤1 year prior to index date	4.49	2.31-8.73	≤.00001	5.17	2.32-11.50	≤.00001	3.82	1.37-10.65	≤.00001	3.31	1.16-9-47	0.025
**Private practice medical specialist**												
10->5 years prior to index date	1.23	1.02-1.49	0.024	1.25	1.02-1.54	0.036	0.97	0.81-1.17	0.746	0.99	0.82-1.19	0.909
5->1 years prior to index date	1.25	1.07-1.47	0.005	1.25	1.06-1.48	0.007	0.84	0.69-1.02	0.073	0.84	0.69-1.02	0.084
≤1 year prior to index date	1.28	1.03-1.59	0.024	1.31	1.04-1.64	0.020	0.79	0.63-0.99	0.048	0.80	0.63-1.01	0.058

CRRs are presented in time intervals prior to the date of dementia diagnosis for cases and compared to the control group of individuals free of dementia at index date.
Abbreviations: MCI: Mild cognitive impairment, CRR: contact rate ratio, CI: confidence interval, GP: general practitioner

Table S5. Sensitivity analysis – Contact rate ratios by level of cognitive decline at diagnosis in secondary care

Secondary care	MCI / Mild dementia						Moderate / severe dementia					
	Unadjusted			Adjusted			Unadjusted			Adjusted		
**Somatic inpatient admissions**	**CRR**	**95 % CI**	**P-value**	**CRR**	**95 % CI**	**P-value**	**CRR**	**95 % CI**	**P-value**	**CRR**	**95 % CI**	**P-value**
10->5 years prior to index date	0.93	0.77-1.13	0.477	0.97	0.81-1.18	0.791	0.99	0.80-1.23	0-911	0.98	0.78-1.22	0.842
5->1 years prior to index date	0.95	0.78-1.15	0.577	0.99	0.82-1.20	0.900	1.33	1.01-1.75	0-043	1.28	0.97-1.70	0.079
≤1 year prior to index date	1.25	0.96-1.63	0.101	1.34	1.03-1.75	0.028	2.26	1.64-3.10	≤.00001	2.17	1.58-2.97	≤.00001
**Somatic outpatient contacts**												
10->5 years prior to index date	1.13	1.03-1.23	0.010	1.11	1.01-1.22	0.037	1.05	0.94-1.16	0.396	1.05	0.94-1.17	0.374
5->1 years prior to index date	1.19	1.09-1.30	≤.00001	1.20	1.09-1.31	≤.00001	1.10	0.98-1.23	0.118	1.13	0.99-1.28	0.061
≤1 year prior to index date	2.14	1.94-2.36	≤.00001	2.25	2.02-2.50	≤.00001	1.65	1.45-1.89	≤.00001	1.71	1.49-1.96	≤.00001
**Somatic emergency admissions**												
10->5 years prior to index date	0.98	0.84-1.14	0.775	1.03	0.88-1.21	0.703	1.35	1.11-1.64	0.002	1.31	1.09-1.59	0.005
5->1 years prior to index date	1.21	1.03-1.42	0.019	1.26	1.08-1.48	0.004	1.54	1.25-1.90	≤.00001	1.49	1.21-1.84	≤.00001
≤1 year prior to index date	1.36	1.03-1.80	0.031	1.41	1.07-1.86	0.014	2.82	2.13-3.75	≤.00001	2.69	2.03-3.55	≤.00001
**Psychiatric inpatient admissions**												
10->5 years prior to index date	0.34	0.12-0.96	0.042	0.30	0.09-0.99	0.048	2.55	1.10-5.89	0.029	2.01	0.93-4.36	0.076
5->1 years prior to index date	0.64	0.21-1.94	0.429	1.96	0.86-4.50	0.111	5.25	1.63-16.91	≤.00001	3.60	1.26-10.28	0.016
≤1 year prior to index date	2.79	0.75-10.30	0.125	6.36	2.60-15.59	≤.00001	19.5	4.72-80.59	≤.00001	14.70	3.70-58.46	≤.00001
**Psychiatric emergency admissions**												
10->5 years prior to index date	0.44	0.15-1.28	0.134	0.31	0.11-0.85	0.024	3.25	1.49-7.08	0.003	2.45	1.20-5.01	0.014
5->1 years prior to index date	0.52	0.15-1.80	0.302	1.70	0.66-4.34	0.270	3.66	1.35-9.89	0.011	3.02	1.20-7.58	0.019
≤1 year prior to index date	2.86	0.91-9.04	0.073	4.55	1.68-12.27	0 003	24.00	7.06-73.30	≤.00001	23.76	7.59-74.38	≤.00001

CRRs are presented in time intervals prior to the date of dementia diagnosis for cases and compared to the control group of individuals free of dementia at index date.
Abbreviations: MCI: Mild cognitive impairment, CRR: contact rate ratio, CI: confidence interval.

Table S6. Sensitivity analysis - Contact rate ratios by type of healthcare contact, stratified by age and sex

Consultation rate ratios (CRR)	Age at index <60 years*			Age at index ≥60*			Male**			Female**		
**Any GP contact**	**CRR**	**95 % CI**	**P-value**	**CRR**	**95 % CI**	**P-value**	**CRR**	**95 % CI**	**P-value**	**CRR**	**95 % CI**	**P-value**
10->5 years prior to index date	1.12	0.97-1.28	0.119	1.09	1.02-1.17	0.011	1.07	0.97-1.19	0.176	1.11	1.03-1.20	0.005
5->1 years prior to index date	1.26	1.11-1.42	≤.00001	1.19	1.11-1.27	≤.00001	1.16	1.05-1.27	0.003	1.23	1.14-1.33	≤.00001
≤1 year prior to index date	1.98	1.74-2.24	≤.00001	1.68	1.57-1.79	≤.00001	1.82	1.67-2.00	≤.00001	1.67	1.56-1.80	≤.00001
**Somatic inpatient admissions**												
10->5 years prior to index date	0.92	0.68-1.25	0.604	0.99	0.84-1.16	0.865	0.82	0.66-1.03	0.083	1.10	0.91-1.33	0.305
5->1 years prior to index date	1.21	0.87-1.67	0.254	1.08	0.90-1.30	0.411	1.01	0.80-1.27	0.918	1.18	0.94-1.48	0.164
≤1 year prior to index date	2.20	1.50-3.25	≤.00001	1.55	1.23-1.95	≤.00001	1.66	1.24-2.24	0.001	1.66	1.27-2.18	≤.00001
**Somatic outpatient contacts**												
10->5 years prior to index date	1.10	0.90-1.34	0.336	1.08	1.81-2.16	0.045	1.06	0.90-1.24	0.487	1.10	1.02-1.19	0.013
5->1 years prior to index date	1.19	1.02-1.39	0.030	1.17	1.07-1.27	≤.00001	1.33	1.14-1.54	≤.00001	1.10	1.02-1.19	0.013
≤1 year prior to index date	2.17	1.72-2.74	≤.00001	1.98	1.00-1.67	≤.00001	2.94	2.51-3.44	≤.00001	1.62	1.48-1.78	≤.00001
**Somatic emergency admissions**												
10->5 years prior to index date	1.00	0.78-1.28	0.996	1.16	1.01-1.33	0.030	1.13	0.94-1.35	0.201	1.13	0.96-1.33	0.136
5->1 years prior to index date	1.34	1.02-1.76	0.038	1.34	1.16-1.54	≤.00001	1.23	1.02-1.49	0.032	1.42	2.30-1.68	≤.00001
≤1 year prior to index date	1.82	1.17-2.82	0.008	1.90	1.53-2.36	≤.00001	2.10	1.56-2.83	≤.00001	1.74	1.34-2.26	≤.00001
**Psychiatric inpatient admissions**												
10->5 years prior to index date	0.37	0.90-1.49	0.161	0.75	0.35-1.61	0.463	0.89	0.40-1.99	0.781	0.71	0.25-2.01	0.514
5->1 years prior to index date	2.08	0.34-12.70	0.426	2.08	1.36-5.74	0.005	1.53	0.53-4.42	0.428	3.64	1.76-7.52	≤.00001
≤1 year prior to index date	12.36	3.46-44.17	≤.00001	8.93	3.47-22.97	≤.00001	12.14	4.64-31.70	≤.00001	8.67	2.99-25.10	≤.00001
**Psychiatric emergency admissions**												
10->5 years prior to index date	0.55	0.08-3.57	0.526	1.02	0.50-2.07	0.959	1.24	0.52-2.96	0.633	0.77	0.30-1.98	0.585
5->1 years prior to index date	1.56	0.37-6.51	0.541	2.23	1.02-4.89	0.044	0.67	0.24-1.87	0.447	3.54	1.62-7.78	0.002
≤1 year prior to index date	9.91	1.58-62.11	0.014	12.12	5.96-24.67	≤.00001	12.64	4.97-32.13	≤.00001	10.03	4.53-22.17	≤.00001

* Adjusted for age, sex (except **), highest attained educational level at age 40 (or at time of diagnosis, whichever came first) and civil status at index date. CRRs are presented in time intervals prior to the date of dementia diagnosis for cases and compared to the control group of individuals free of dementia at index date.

Sensitivity analysis presented for a selection of types of contacts. Remaining types of contacts are equally similar to the results found in the main analysis.

Abbreviations: CRR: contact rate ratio, CI: confidence interval. GP: general practitioner
